# Calciphylaxis of the Upper Limbs—A Rare and Serious Disease with Multidisciplinary Treatments—A Case Series and Literature Review

**DOI:** 10.3390/diagnostics15091179

**Published:** 2025-05-06

**Authors:** Mihaela Pertea, Malek Benamor, Andra-Irina Bulgaru-Iliescu, Abderrazek Abid, Said Abid, Alexandru-Hristo Amarandei

**Affiliations:** 1 Department Plastic Surgery and Reconstructive, Faculty of Medicine, “Grigore T. Popa” University of Medicine and Pharmacy, 700115 Iasi, Romania; mihaela.pertea@umfiasi.ro (M.P.); bulgaru-iliescu_andra-irina@d.umfiasi.ro (A.-I.B.-I.); 2 Department of Plastic Surgery and Reconstructive Microsurgery, “Sf. Spiridon” Emergency County Hospital, 700111 Iasi, Romania; alexamarandei@yahoo.com; 3 Research Laboratory of Oral Health Rehabilitation, Monastir University, LR12ES11, Monastir 5000, Tunisia; 4 Trauma and Orthopedics Department, Faculty of Medicine, Fattouma Bourguiba University Hospital, University of Monastir, 1st of June Street, Monastir 5000, Tunisia; abderrazek.abid.2@gmail.com (A.A.); saidabid94@gmail.com (S.A.)

**Keywords:** calciphylaxis, upper limb, end-stage renal disease, dialysis, surgery

## Abstract

**Background/Objectives:** Calciphylaxis or calcific uremic arteriolopathy is a rare but highly lethal pathology that occurs most frequently in a uremic context, although it can also occur outside of this context. It is characterized by the appearance of necrotic skin lesions. Localization to the upper limbs is rare and has a similarly progressive evolution. **Case Presentation:** We present a series of two cases—a male and a female—with calciphylaxis diagnoses (including biopsies) and with the patients undergoing dialysis for end-stage renal disease, both with infected and extensive necrotic lesions to the hands and fingers. Both cases required serial debridement treatments and amputations. A literature review was conducted using the precise search terms “calciphylaxis”, “upper limb”, “uremic calcific arteriolopathy”, and “end-stage renal disease” from January 2010 to May 2024. One of the two reported cases ended with the patient’s death. The results of the literature review (comprising seven similar cases) confirmed the rarity of calciphylaxis lesion localization to the upper limbs and the high mortality rate among these patients despite administered treatments. No therapeutic protocol for these cases was confirmed. **Conclusions:** The treatment of calciphylaxis cases is multidisciplinary. Although surgical intervention is controversial, it is necessary in some cases, sometimes serially. Localization to the thoracic limbs has the same evolution and poor prognosis as other localizations. A standardized therapeutic protocol for these cases is still far from being established.

## 1. Introduction

In 1961, following experiments conducted on laboratory animals in which vascular calcifications were induced through hyperparathyroidism and hypervitaminosis D, Hans Selye defined the condition known as calciphylaxis [1]. The condition is also known as uremic calcific arteriolopathy. This disease is most commonly associated with chronic kidney disease (CKD) and is diagnosed predominantly in patients with end-stage renal disease (ESRD). However, cases of calciphylaxis have also been reported in patients with normal renal function [1,2]. Calciphylaxis is frequently documented in the literature among patients with terminal-stage renal disease undergoing dialysis (primarily hemodialysis). These patients often present with multiple comorbidities, including hypertension, obesity, insulin-dependent diabetes, liver disease, and cardiac conditions [3,4]. The etiology and pathogenesis of the disease remain poorly understood and unclear. Clinically, calciphylaxis is characterized by the appearance of subcutaneous nodules that are firm and painful and are initially surrounded by erythematous areas that later develop into violaceous discoloration resembling livedo reticularis. These lesions subsequently progress to necrotic eschars, ulcerations, or gangrene. Calciphylaxis lesions can involve skeletal and cardiac muscles, as well as joints, lungs, eyes, penises, breasts, pancreas, and intestines [5,6,7]. The most common sites of involvement are the lower extremities, accounting for 71% of cases reported in the literature [8,9]. This is followed by the gluteal region and thighs, typically in areas with well-developed subcutaneous adipose tissue. The lesions are often symmetrical. Reports of calciphylaxis affecting the upper extremities are rare, and unilateral cases in this region are even less frequently documented [10,11]. The estimated annual incidence of calciphylaxis ranges from 1 to 35 cases per 10,000 dialysis patients worldwide [10]. This incidence may be underestimated due to undiagnosed cases, often resulting from a lack of awareness about the disease [10,11,12]. The described risk factors associated with the development of calciphylaxis include undergoing hemodialysis, being of female sex, and presenting with obesity, diabetes, hypercalcemia with hyperphosphatemia, hypoalbuminemia, autoimmune diseases, and elevated alkaline phosphatase levels, as well as undergoing treatments with warfarin, calcium, vitamin D, iron, and para-thyroid hormone, medications administered in various forms of presentation from different manufacturers. Regarding warfarin treatment, studies have identified an incidence of calciphylaxis among patients treated with warfarin of 6.24 per 1000 patient-years compared to 3.41 per 1000 patient-years in those not receiving this medication [8,13,14]. The presence of calciphylaxis in patients with chronic kidney disease is associated with poor prognoses and high mortality rates. Some studies have reported a one-year mortality rate of approximately 50% following diagnosis, while others have indicated mortality rates ranging from 30% to 80% in cases with ulcerative lesions and multiple comorbidities [15,16]. In cases of calciphylaxis diagnosed in non-uremic patients, the one-year mortality rate has been reported at 52% [15,17]. In these patients, calciphylaxis can occur in the context of primary hyperparathyroidism, oncologic conditions treated with chemotherapy, or autoimmune diseases [11]. The primary cause of death in nearly all cases is generalized sepsis. However, it has been reported that the presence of lesions confined to the extremities is associated with better survival rates [7,18]. Histological examinations have typically revealed vascular calcifications accompanied by intimal hyperplasia and microthrombosis [8,19].

The treatment of calciphylaxis is complex and multifaceted. Early diagnosis is paramount for limiting cutaneous lesion progression, making it the most critical step in management [20,21,22]. Once cutaneous lesions appear, their monitoring and care, prevention of infection, elimination of potential risk factors (such as discontinuing warfarin, iron, or calcium therapies), appropriate treatment and management of comorbidities, ensuring proper hydration and nutrition, and monitoring and addressing renal disease are essential components of care [23,24]. Local treatment through debridement remains controversial, as there is no standardized timing or extent for surgical intervention. In some cases, even a diagnostic biopsy may lead to extensive necrosis, with rapid progression [24]. Correcting calcium and phosphate levels is critical. Recently, the literature has highlighted the efficacy of therapies such as sodium thiosulfate, bisphosphonates, and hyperbaric oxygen as treatment options for these cases [25,26].

We present a series of two cases: one female patient and one male patient, both diagnosed with calciphylaxis. Both patients had chronic kidney disease, were undergoing hemodialysis, and exhibited multiple comorbidities associated with known risk factors. In both cases, the patients presented with localized infections of the hands, unilaterally, accompanied by general deterioration due to local and systemic septic statuses. Surgical interventions were deemed absolute and urgent indications in both instances. The definitive diagnoses were confirmed through histopathological examination. The outcomes in both cases were poor, with the patients ultimately succumbing to their conditions. The significance and rarity of calciphylaxis cases localized unilaterally to the upper limbs are supported by a systematic literature review conducted over a 10-year period, which complements this study.

## 2. Case Presentation

### 2.1. Case Series

A series of two cases admitted and treated in the Plastic Surgery Department of St. Spiridon Hospital in Iasi were included in this study. In both cases, informed consent was obtained and signed by the patients. The study received approval from the hospital’s Ethics Committee (approval no. 127/17 December 2024) for reporting purposes. Data for the study were collected from the patients’ medical records and entries in the hospital’s electronic information system.

#### 2.1.1. Case Report 1

**Figure 1:**
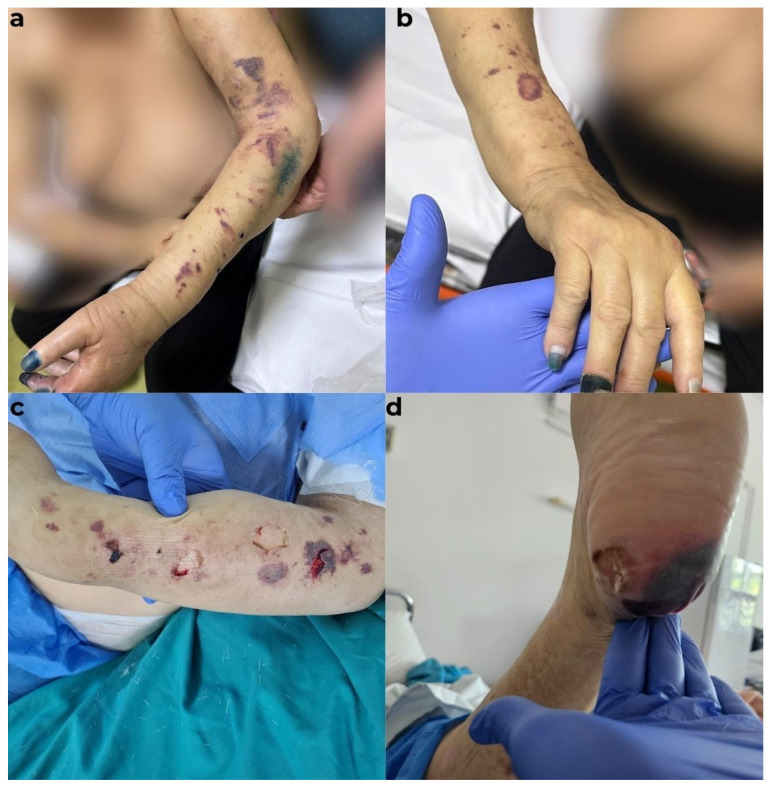
Cutaneous aspects at admission. (**a**) Disseminated ecchymoses on both upper limbs, with digital necrosis involving the second and third fingers of the left hand. (**b**) Periungual necrotic areas on the third and fourth fingers of the right hand. (**c**) Detailed view of cutaneous lesions on the left forearm (with an arterio-venous fistula). (**d**) Necrotic area on the calcaneal region of the left foot.

A 70-year-old female patient with a known history of chronic kidney disease, on a hemodialysis program for the past five years, presented with a palmar abscess accompanied by a small fistulized area. The overlying skin was warm, erythematous, and hypoesthetic, with palpation revealing a fluctuant region characteristic of a lesion. Additionally, necrotic skin areas were observed at the levels of the second and third phalanxes of the index finger and the distal phalanx of the middle finger of the left hand, as well as at the distal phalanxes of the fourth and fifth fingers of the right hand. Necrotic areas were also noted in the calcaneal and pre-Achilles regions of the right leg and foot. On both forearms and legs, there were ecchymoses and necrotic skin lesions of varying sizes, with diameters ranging from 0.5 to 3 cm, interspersed with areas of epithelial loss. The patient reported no history of trauma during the anamnesis (Figure 1).

The patient had a history of comorbidities, including chronic heart failure, insulin-dependent diabetes mellitus, hypertension, atrial fibrillation, and mixed anemia. The arterio-venous fistula used for hemodialysis was located in the upper third of the left forearm (on the same side as the palmar infection). The hemodialysis regimen was set at three times per week. At the time of admission, the patient had been severely limited in mobility, although the anamnesis indicated that she had not previously experienced this level of impairment prior to her hospital presentation.

**Figure 2:**
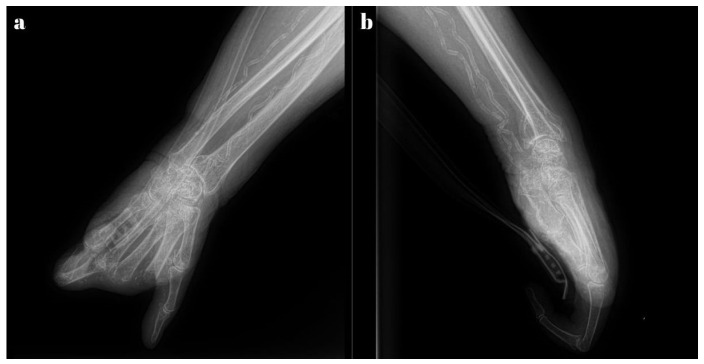
Radiological exam—arteries with calcifications in calciphylaxis: (**a**) lateral view, and (**b**) anterior-posterior view.

Among the subjective complaints, the most significant symptom was pain, characterized by its onset in an ischemic context. The radiological examination revealed an old malunited distal radius fracture, arthritic changes in the left wrist, osteoporosis, and calcifications in the walls of the radial and ulnar arteries (Figure 2).

An echo-Doppler exploration of the left radial and ulnar arteries showed the presence of arterial flow in both vessels. The general status of the patient during the emergency visit did not allow for vascular angiography (CTA/MRA). At admission, the patient’s biological status was poor (leukocytes: 21.99 × 10^3^/µL (N = 4.0–10.0/×10^3^/µL), C-reactive protein: 18.02 mg/dL (N = 0–0.5 mg/dL), glucose: 88 mg/dL (83–110 mg/dL), ionized calcium: 4.04 mmol/L (3.82–4.82 mg/dL), total calcium: 8.43 mmol/L (N = 8.80–10.00 mg/dL), and eGFR: 9.16 (N => 90 mL/min/1.73 m^2^)).

**Figure 3:**
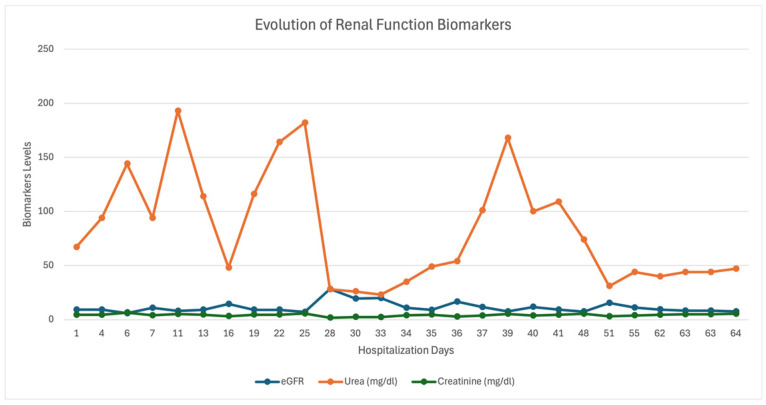
Renal function evaluation results during hospitalization.

**Figure 4:**
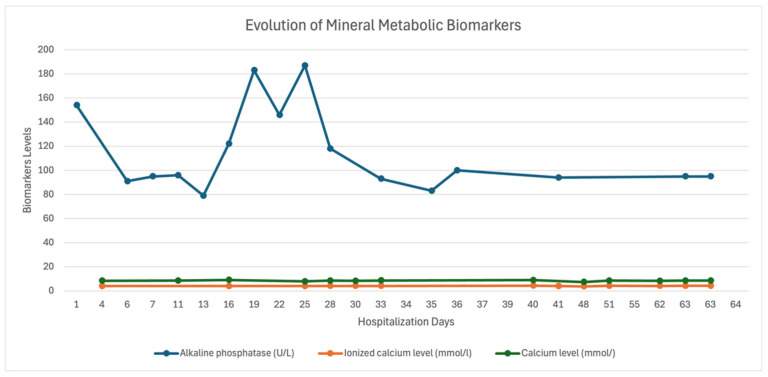
Results of the mineral metabolism evaluation during hospitalization.

The progression of biological parameters during hospitalization fluctuated (Figure 3 and Figure 4).

**Figure 5:**
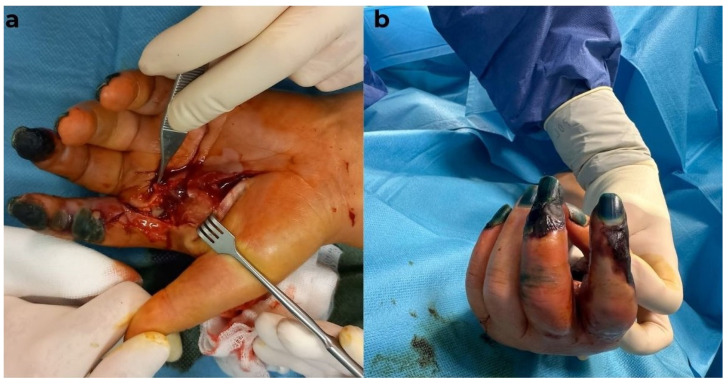
Intraoperative view: (**a**) the time of opening the abscess in the left hand, and (**b**) a dorsal view of the left hand (digital necrosis at the levels of the second and third fingers).

Due to the patient’s deteriorated general condition, abnormal biological parameters, and local appearance, the indication for emergency surgical intervention under loco-regional anesthesia (infraclavicular block) was established. Surgical intervention was performed, during which secretions were collected for microbiological examination, followed by evacuation of the secretion and abundant lavage with antiseptic solutions. The necrotic segments at the levels of the second and third fingers were preserved, as the patient expressed disagreement with any amputation procedure (Figure 5).

All surgeries were performed meticulously to minimize tissue trauma, as the diagnosis of calciphylaxis was suspected from admission given the patient’s seven-year history of chronic kidney disease. Biopsy samples were collected from the soft tissues for histopathological examination to confirm the diagnosis. Empiric antibiotic therapy was initially initiated until the identification of the pathogen in the wounds. The patient’s general condition deteriorated, and she was transferred to the intensive care unit (ICU). The secretions collected tested positive initially for *Klebsiella pneumoniae* and *Candida* spp., and the infectious disease specialist, in agreement with the nephrologist, recommended antibiotic treatment. The patient’s general condition remained compromised, and the dialysis session was postponed by one day, as per the nephrologist’s decision. During this period, the patient received appropriate treatment for her cardiac conditions and insulin therapy as per the recommendations of the endocrinologist

**Figure 6:**
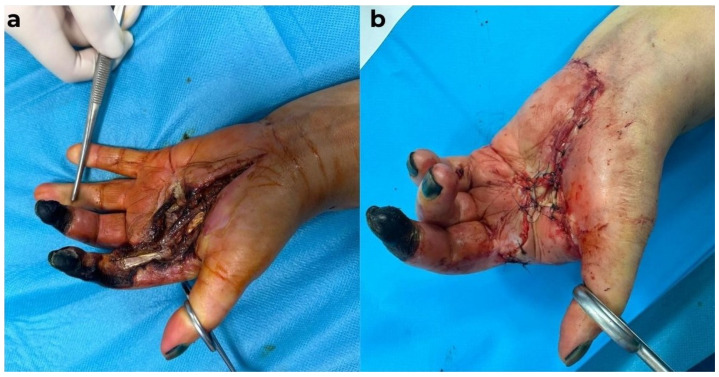
Intraoperative view: (**a**) extensive palmar and digital necrosis compared to the initial admission, and (**b**) view after debridement.

Given the patient’s critical condition, a second surgical intervention was performed. After obtaining consent for the amputation of only the second digit, the procedure was carried out accordingly, following a thorough chemical debridement. The remaining defect was covered with a flap from the amputated finger. As before, all surgical maneuvers were performed gently, with minimal tissue trauma, while the patient simultaneously received targeted antibiotic therapy and proper rebalancing in the ICU (Figure 6).

During this time, the disseminated cutaneous lesions on both forearms and the left upper arm, as well as those on the calcaneal region, were treated conservatively with local debridement, antiseptic solutions, and epithelializing creams. The patient was placed back on a regular hemodialysis schedule. On the seventh day of disease progression, the patient’s general condition worsened, with confusion, apathy, and difficulty speaking, raising suspicion for a stroke, which was ruled out by CT examination

Locally, the progression was unfavorable, with necrosis of the flaps. As a result, a second surgical intervention was performed, leading to the amputation of the third and fifth digital rays, for which consent was obtained from the patient. During hospitalization, secretions were collected from the wounds and antibiotic treatment was adjusted following the recommendation of the infectious disease specialist and in agreement with the nephrologist. The cultures tested negative on the 10th day of hospitalization but became positive again for *Klebsiella pneumoniae* and *Proteus mirabilis* on the 17th day. On the 45th day, contamination with *Acinetobacter baumannii* was identified. In this case as well, appropriate treatment was administered.

**Figure 7:**
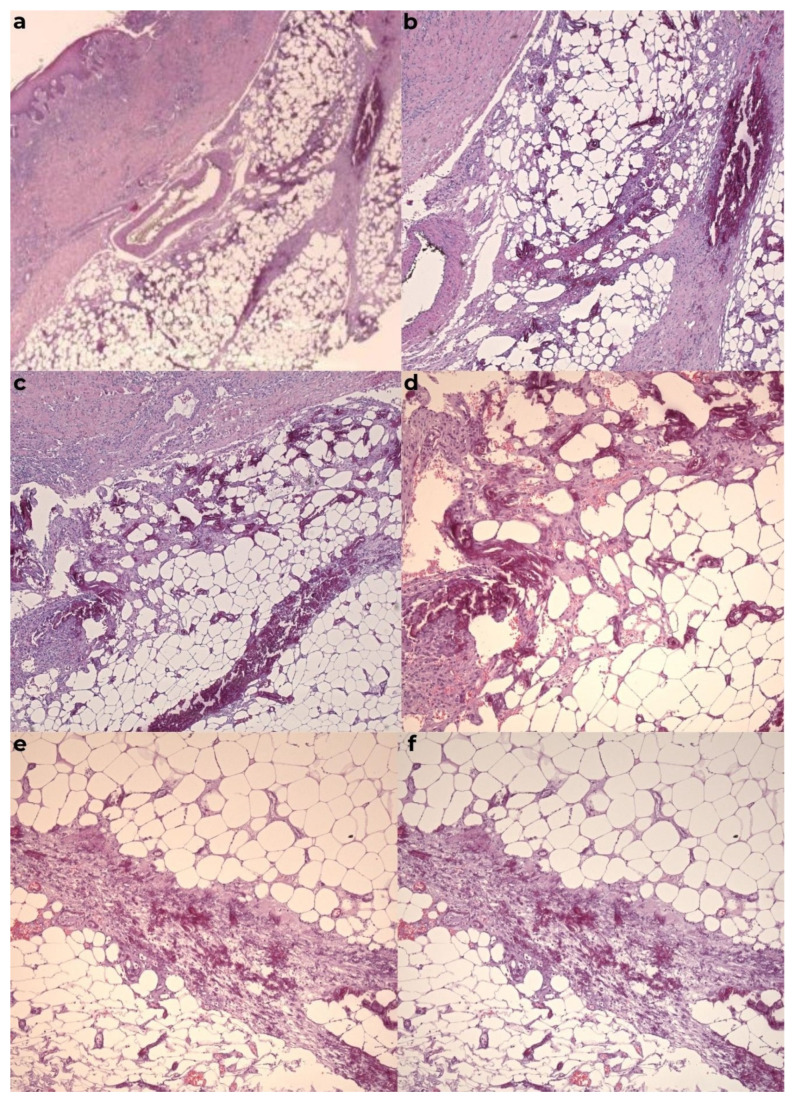
The calcified material was surrounded by multinucleate giant cells, often associated with psammoma-like calcifications. Calcium deposits were localized in the intima and media of the small and large vessels in the deep dermis and hypodermis and were frequently accompanied by vascular thrombi. Hemorrhages may have occurred in the subcutaneous adipose tissue, along with liponecrosis and chronic lymphoplasmacytic inflammatory infiltrates. Calcium deposits could also be observed in the fibrous septa of the subcutaneous adipose tissue. (**a**) HE×2.5, (**b**) HE×5, (**c**) HE×5 ob0001, (**d**) HE×10, (**e**) HE×10 ob0001, and (**f**) HE×10 ob0004.

**Figure 8:**
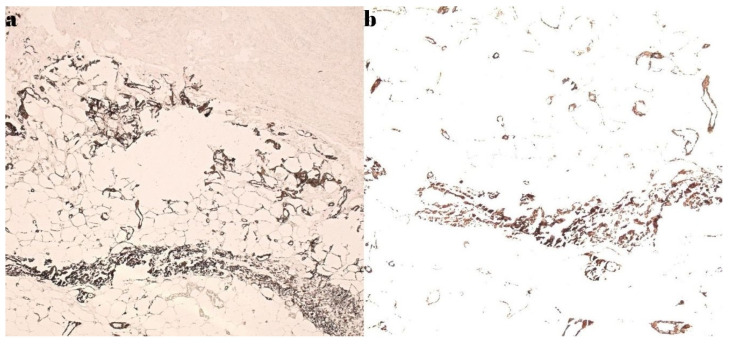
Special staining: (**a**) von Kossa ×5, and (**b**) von Kossa ×10, with the calcium deposits seen in calciphylaxis.

The histopathological examination confirmed the diagnosis of calciphylaxis, revealing a moderately infiltrated polymorphous inflammatory response in the dermis, marked by congestion and edema. The venous vessels in the deep dermis and subcutaneous tissue were dilated, with intraluminal fibrino-hemato-leukocytic thrombi, while the arterial vessels displayed thickened walls with intimal hyperplasia obstructing the lumen. Additionally, there was prominent sclero-hyalinosis in the subcutaneous tissue and delineating lobules of adipose tissue, with some exhibiting features of liponecrosis, along with isolated dystrophic calcifications. These morphological features were consistent with ischemic changes secondary to calciphylaxis. Routine hematoxylin-eosin staining, as well as special stains such as von Kossa, were performed (Figure 7 and Figure 8).

The patient was informed that the disease would progress, potentially requiring amputation of healthy tissue from the forearm. At that time, the patient and her family did not agree to any further form of amputation. However, the local progression remained unfavorable in terms of tissue viability, despite the negative results from the secretions. Serial debridement treatments were performed, and negative pressure therapy was applied to the remaining defects to promote granulation, though without the desired results. Throughout the hospitalization, the patient received hemodialysis and treatment supervised by the nephrologist. Despite all the efforts of the general multidisciplinary treatment and the local treatment for covering the remaining skin defects, no long-term surgical solution was found. Therefore, in agreement with the patient, a necessary amputation was decided to prevent any possibility of infection. On the 62nd day of hospitalization, following a multidisciplinary preoperative evaluation, a necessary amputation was performed at the distal third of the forearm. However, on the second postoperative day, the patient experienced a cardiac arrest, which, unfortunately, was irrecoverable.

#### 2.1.2. Case Report 2

**Figure 9:**
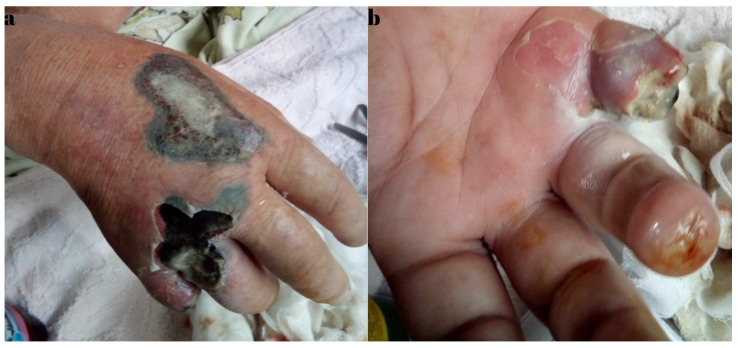
Local aspect before admission. (**a**) dorsal view: necrosis on the dorsal side of the right hand with erythema around the lesion and edema, and (**b**) Palmar view: wet necrosis at the amputation stump of the fourth and fifth fingers.

A 57-year-old male patient presented for a consultation due to pain in his right hand, with a necrotic area on the dorsal surface of the hand and cellulitis, as well as wet gangrene at the level of the fifth finger, with an amputation stump from two months prior. Additionally, there was an amputation stump at the distal level of the fourth finger, which had been performed at another medical facility. Changes in color and vascularization were observed at the level of fingers two to four, with erythema and generalized edema of the hand (Figure 9).

**Figure 10:**
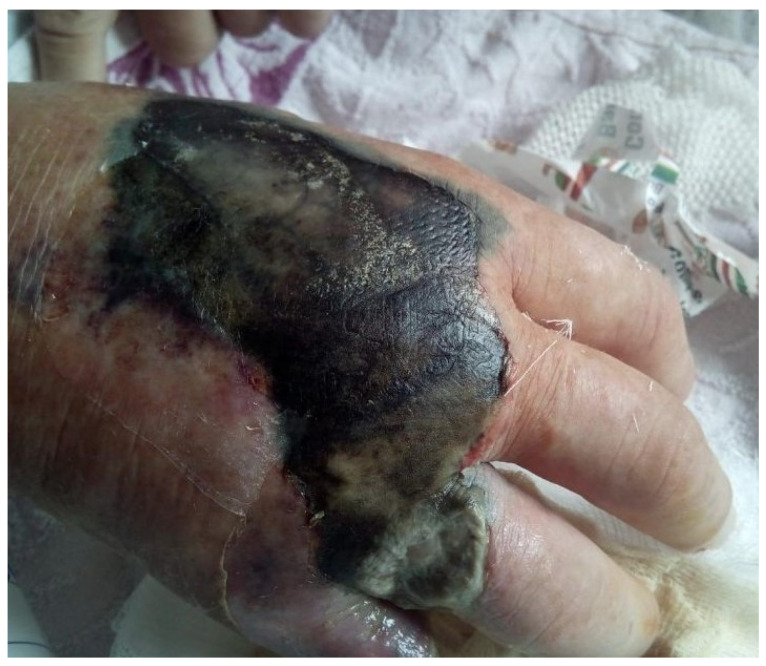
Second admission view: extension of the wet necrosis on the dorsal side of the right hand.

Emergency hospitalization was recommended. The patient refused hospitalization and returned after one week with an aggravated local status and extension of the necrotic areas and worsening of the general condition (Figure 10).

**Figure 11:**
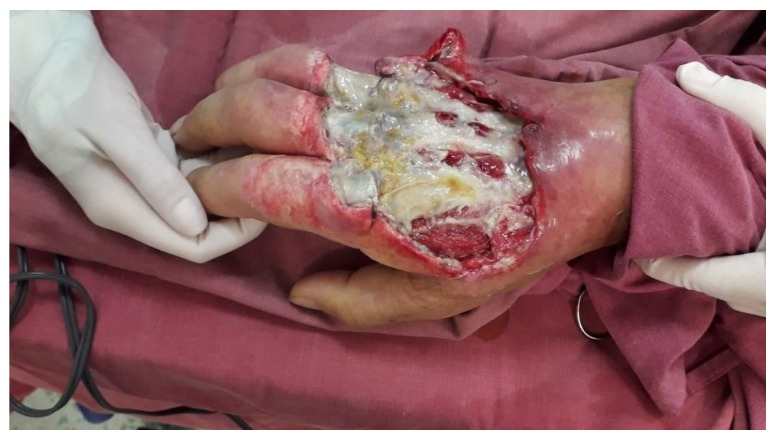
Intraoperative view with exposure of the extensor tendons and devitalized tissues after negative pressure therapy.

The patient, known to have stage V chronic kidney disease, had been on hemodialysis for 4 years and presented with the following numerous comorbidities: insulin-dependent type 2 diabetes mellitus, bilateral thigh amputations performed 1 year prior due to obliterative arteriosclerosis, secondary hypertension, and mixed anemia. The biological constants upon admission are as follows: WBC = 23.56 × 10^3^/µL (N = 4.0–10.0 × 10^3^/µL), HGB = 9.7 g/dL, CRP = 31.47 mg/dL (N = 0.00–0.50 mg/dL), glucose = 217 mg/dL (N = 74–106 mg/dL), total calcium = 10.58 mg/dL (8.60–10.00 mg/dL), urea = 57 mg/dL (N = 17–49 mg/dL), creatinine = 4.73 mg/dL (N = 0.50–1.20 mg/dL), and presepsin = 4392 pg/mL (increased risk if over 1000 pg/dL). Surgical indication was established under regional anesthesia, with secretion collection from the apparently infected areas, debridement, and abundant lavage with antiseptic solutions. The surgical maneuvers were performed with careful consideration, as calciphylaxis was suspected from the start. The bacteriological exam revealed the presence of *Klebsiella pneumoniae*, which led to the initiation of targeted antibiotic therapy, guided by the infection specialist. Despite local and general treatment, the patient’s condition worsened, and the biological constants changed negatively. On the fourth day of hospitalization, the patient’s biological constants were as follows: WBC = 25.94 × 10^3^/µL, HGB = 8.3 g/dL, CRP = 30.12 mg/dL, glucose = 168 mg/dL, urea = 240 mg/dL, and creatinine = 8.38 mg/dL. In this biological, local, and general context, a second surgical intervention was decided, during which debridement was completed, and the amputation of the fifth digital ray at the metacarpophalangeal joint was performed, followed by the application of negative pressure therapy on the remaining skin defects with exposure of extensor tendons and devitalized tissues (Figure 11).

**Figure 12:**
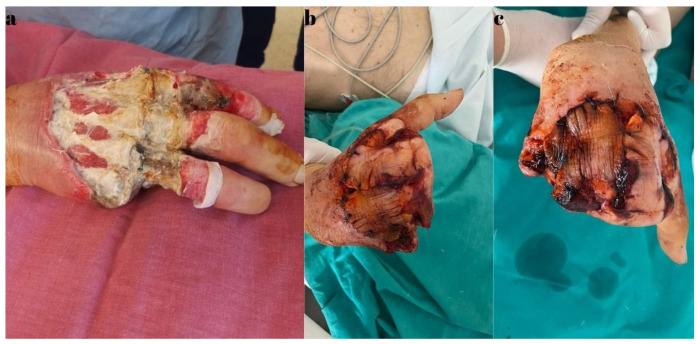
(**a**) Progression of the tissue devitalization. (**b**,**c**) An amputation view after 3 days, with the development of marginal necrosis areas on the flaps.

During hospitalization, the biological constants showed variable fluctuations, but the patient’s general condition remained stable. Throughout this period, the patient underwent hemodialysis, with a schedule of once every two days, under the supervision of a nephrologist. Locally, there was progressive extension of the necrotic areas on the dorsal side of the hand, and intraoperative, vascular calcifications were observed macroscopically. Biopsy samples were collected for histopathological examination. As a result of the extensive necrosis, a third surgical intervention was required on the 14th day of hospitalization. A necessary amputation of the fifth ray (including the metacarpal), two-thirds of the fourth metacarpal, and one-third of the third metacarpal bone was performed, followed by covering the remaining defects on the dorsal side and ulnar border of the right hand with flaps harvested from the amputated fingers (Figure 12).

The patient’s high blood pressure throughout the hospitalization required periodic consultations and administration of antihypertensive medications in varying doses as indicated by a cardiologist. The patient, diagnosed with a depressive syndrome, also received psychiatric support throughout the hospitalization. The evolution was favorable, with a trend toward normalization of the biological constants and inflammatory markers. The patient’s local status was slowly favorable, with secretion from the wounds becoming negative under targeted antibiotic treatment, as recommended and managed by the infectious disease specialist based on microbiological results and repeated antibiograms during hospitalization. The histopathological examination also confirmed extensive calcification and vascular thrombosis lesions, which, correlated with the clinical context, confirmed the diagnosis of calciphylaxis. A suffering of the flaps was identified after the last surgical intervention but without rapid progression. In these conditions, the patient requested discharge, understanding and assuming the risks involved. Follow-up care after discharge was possible only for two weeks, after which the patient did not return to our service. In both cases presented, the dialysis parameters were recorded in the dialysis center where each patient was followed and dialyzed. Also, the second patient’s medical history did not reveal any significant disturbances in his calcium-phosphate metabolism; therefore, no specific treatment was indicated.

## 3. Discussion

Unfortunately, uremic calcific arteriolopathy diagnosed in uremic patients, with a reported prevalence of 4%, also occurs in non-uremic patients. Its pathophysiology is still debated, and it lacks a standardized treatment. The evolution of this condition often results in a negative prognosis, especially when known risk factors are present [7]. It can be diagnosed in patients without renal disease but with conditions such as oncological diseases treated with chemotherapy, primary hyperparathyroidism with normal renal function (Marques), liver cirrhosis, and autoimmune diseases [1,11]. In all reported cases, including the present study, the importance of risk factors has been emphasized, including the following: hemodialysis, comorbidities such as arterial hypertension, diabetes mellitus, ischemic heart disease, and obesity, to which can be added local trauma, hypercalcemia, and hyperphosphatemia, being of Caucasian race, coagulation disorders, and medication with warfarin [11]. The cutaneous lesions in calciphylaxis can be described as having an evolution that begins with hard and painful subcutaneous nodules and erythematous areas, followed by the appearance of ischemic red-violaceous zones that progress to necrosis. These lesions often become infected due to a patient’s general status; frequently, a patient presents already in an infected stage [8,27,28]. The first and most important symptom is pain of varying intensity [11]. In one of the cases in the present study, the cutaneous lesions were distributed across the entire body surface and were present on both hands’ fingers and forearms bilaterally, as well as the thorax, abdomen, and lower limbs. In the other case, the lesions were unilateral, which represents a less common situation. Some authors have correlated the localization of lesions with mortality rates, showing that localization at the level of the shoulder or arm up to the elbow (proximal upper limb) is associated in 50% of cases with a poor prognosis, reporting a mortality rate of 70% [29,30,31]. The mortality rate in cases where calciphylaxis lesions are diagnosed on the lower limbs is 33.5%, while the presence of cutaneous lesions of calciphylaxis on the forearms and hands has a recorded mortality rate of 46.9% in most cases. In the current study, one of the two cases ended with the patient’s death (50%), although the final outcome of the second case was not known as the patient did not return for follow-up [31,32]. In both cases presented, surgical intervention did not lead to a positive outcome in the overall progression of the patients’ conditions. The extensive necrosis characteristic of this condition means that, although an initial necrosectomy is performed on viable tissue, subsequent widespread lesions often necessitate additional necrosectomies and debridement treatments—each carrying risks of infection and other complications. These lesions typically require multiple staged necrosectomies and debridement treatments.

When necessary, a high-level amputation, such as at the forearm, may be considered a more effective option. However, it is often difficult for surgeons to propose—and for patients to accept—such a major amputation, especially if there is still a chance for a more limited procedure. There are situations, as illustrated in the first case, where the patient is unwilling to undergo even an amputation of a clearly necrotic finger. There is no guarantee that an early, high-level amputation would alter the course or prognosis of the disease, though in some cases it might be justified as a life-saving measure.

We conducted a systematic search in the PubMed database and on Web of Science and Scopus utilizing the precise search terms “calciphylaxis”, “upper limb”, “uremic calcific arteriolopathy”, and “end-stage renal disease”. The search was specifically confined to case reports published from January 2010 to May 2024 and found seven similar articles available in the literature. We studied reports in English (Table 1).

In the literature, we found five cases (71.42%) that reported female patients. The ages of the patients in the studied cases ranged from 31 to 75 years, with a mean age of 55 years. All patients with end-stage renal disease (ESRD) who had been on hemodialysis for over a year had associated comorbidities included in their risk factors. In one of the cases, the patient was receiving chronic warfarin treatment [37]. Only one study did not specify the associated diseases [34]. In the reported cases included in the systematic review, regarding the comorbidities in the studied cases, diabetes was present in three cases (42.85%), hypertension in three cases (those who also had diabetes), ischemic heart disease in two cases (28.57%), and in one case (14.28%), hypothyroidism and an unspecified collagen disease were recorded. The main symptom reported in all cases was pain, usually described as severe, followed by changes in color, cyanosis, livedo reticularis, ischemia, necrosis, and dry gangrene at the skin level. The skin lesions were present at the level of the fingers in five cases, one of which was associated with similar lesions on the toes. In two of the cases (28.57%), the skin involvement was at the level of the arms, one of which was also associated with lesions on the breast and abdomen, and the other with gluteal lesions. Regarding the localization of the lesions on the fingers, only one case specified that there was bilateral involvement of the characteristic calciphylaxis skin lesions. The diagnosis was established based on the clinical appearance and the renal disease context, with four cases confirming the presence of vascular calcifications through radiological examination. In six out of the seven cases, histopathological examinations of the biopsy samples were performed. Only one case mentioned the absence of biopsy in establishing the diagnosis of calciphylaxis [10]. Regarding treatment, it was both medical and surgical in some cases. In three cases, the administration of sodium thiosulfate was specified, and in two cases, it was combined with Cinacalcet, which, in turn, was associated in one case with apixaban. In three cases, there were no records of surgical treatment. In one case, parathyroidectomy was performed [33], associated with serial debridement treatments, followed by coverage of the remaining skin defects with split-thickness skin grafts. In this case, the patient died 4 months after the diagnosis was established. In three cases, a conservative treatment approach was chosen for the skin lesions, with the main goal of preventing their infection. The evolution was unspecified in four cases. In the other three cases, the patient passed away four months after diagnosis in the first case, six weeks later for the second case, and in the last case, the time of death was unspecified. None of the reported cases in the literature mentioned healing of the skin lesions.

Although the method of establishing a diagnosis is not well established, the appearance and evolution of local lesions along with the primary symptom—pain—in the context of a patient with kidney disease leads to a presumptive diagnosis of calciphylaxis, which some authors have considered sufficient [15,23]. Radiological examination with visualization of the calcified arterial axes (similar to the cases presented) is not specific, according to some authors, but can be considered in establishing a diagnosis [7,38,39]. In these cases, even the importance of a skin biopsy in the diagnostic assessment is controversial, with some authors believing that an anatomopathological diagnosis is necessary [8], while others think it only helps, as there are no pathognomonic lesions of the disease [40]. In the reports in this study, in both cases, the result of the pathological examination with usual and special stains (von Kossa) established the diagnosis of calciphylaxis in the clinical context. Regarding treatment, there are recommendations in the literature, but no well-established protocol exists. There are several directions that should be followed, as follows: pain management, stabilization of risk factors, renal disease management, discontinuation of treatments that favor the appearance and maintenance of calciphylaxis lesions (such as warfarin), normalization of Ca and P levels, and medical or surgical treatment of parathyroidism [11,31,41]. From the perspective of local calciphylaxis lesions, care is recommended for these lesions and the surrounding unaffected skin, with the primary goal of preventing infection and treating, limiting, and sanitizing it when present. Some authors have recommended avoiding surgical debridement interventions and using different types of hydrocolloid and hydrogel dressings for the treatment of cutaneous lesions, associated with targeted antibiotic treatment [1,42]. There are cases, similar to the two case reports herein, where patients have presented with acute infections, as follows: phlegmon, abscess, and infected lesions with cellulitis, where surgical intervention is necessary. In these cases, primary surgical debridement is followed by others in series to complete the debridement treatment of areas that progressively devitalize in the context of the disease [33]. Infection treatment at the wound level is difficult, requiring targeted antibiotic therapy that is also nephrotoxic in the context of severe renal impairment [43]. The literature has noted the presence of germs such as *Staphylococcus aureus* and *Acinetobacter baumannii* [11]. In the two reported cases, *Klebsiella pneumoniae* was detected upon admission, with one case associated with *Candida* spp.

It has been noted in the literature that sodium thiosulfate treatment was used in cases for calcium chelation, having a vasodilatory effect and endothelial regeneration [26,44], as well as bisphosphonates and hyperbaric oxygen therapy at the wound level [11]. For the treatment of hyperparathyroidism, parathyroidectomy or the use of an oral calcimimetic like Cinacalcet is recommended [31,45]. In the cases presented, the patients did not benefit from any of these treatments.

The entire treatment and therapeutic management of these patients could not be performed directly and completely without close interdisciplinary collaboration in a mixed team [46]. In the two reported cases, the following numerous specialties were involved: plastic surgeons, intensivists, nephrologists, infectious disease specialists, cardiologists, diabetologists, anatomopathologists, and psychologists.

## 4. Conclusions

Calciphylaxis or calcific uremic arteriolopathy is a severe disease with a poor prognosis and high mortality rate, frequently occurring in patients with end-stage renal dis-ease that are undergoing dialysis. Monitoring the appearance of cutaneous lesions in these patients is essential for limiting their occurrence or at least diagnosing them in early, non-infected stages. Once necrotic areas appear, preventing their infection is crucial. The onset of severe infections in these patients necessitates surgical treatment, although it is controversial and carries significant risks. The progression of necrotic lesions requires serial debridement treatments and even amputation of the affected limb, and often, no single surgical technique can achieve definitive healing of the cutaneous lesion. Multidisciplinary team management of these patients is essential for obtaining the best possible outcome for each case individually.

## Figures and Tables

**Table 1:** Registered studies and their analysis.

	Author/ Year	Sex	Age	Location	Symptoms	Diagnosis	Other Diseases	Treatment	Evolution
1	Stavros K. 2014 [33]	M	46	arms and gluteal region	pain, myalgia, bruises, and pressure sores	clinical exam and biopsy	ESRD and HTN	parathyroidectomy, multiple surgical debridement treatments, and skin grafts	death 4 months after diagnosis
2	Kazanji N. 2015 [34]	F	31	D3RH	pain, livedo reticularis, cyanosis, and pressure sores	clinical exam, biopsy, and X-ray	unspecified	sodium thiosulfate and surgical treatment—unspecified	unspecified
3	Kirby L. 2016 [35]	F	68	arms, breast, and abdomen	unspecified	clinical exam and biopsy	ESRD	analgesics and conservative treatment for skin lesions	death 6 weeks after diagnosis
4	Abbas Hartoon 2019 [36]	F	32	D2RH and D3LH	pain, edema, and skin color changes	clinical exam and biopsy	ESRD	Sodium thiosulfate, cinacalcet, sevelamer care of skin lesions to prevent infection and surgical treatment—unspecified	unspecified
5	Ko Harada 2019 [37]	M	69	D3 and 4RH	pain, ischemia, and necrosis	clinical exam, X-ray, and biopsy	ESDR, DM, and collagen disease	discontinuation of warfarin treatment and conservative treatment of skin lesions	unspecified
6	Bielejewska A. 2020 [11]	F	64	fingers, hands, and toes	pain, ischemia, and necrosis	clinical exam, biopsy, and X-ray	ESRD, DM, HTA, ischemic heart disease, and ATS	Cinacalcet, Apixaban, sodium thiosulfate, and surgical treatment—left upper limb amputation	death
7	Zay Yar Aung 2022 [10]	F	75	D5LH, D4, and 5RH	dry gangrene	clinical exam and X-ray (no biopsy)	DM, HTN, ischemic heart disease, and hypothyroidism	unspecified (no surgery)	unspecified

## Data Availability

The original contributions presented in the study are included in the manuscript; further inquiries can be directed to the corresponding authors.

## Abbreviations

The following abbreviations are used in this manuscript:

CUA	calcific uremic arteriolopathy
CHD	chronic kidney disease
ESRD	end-stage renal disease
M	male
F	female
Rx	radiological exam
HTN	hypertension
D	digit
LH	left hand
RH	right hand
ATS	atherosclerosis
DM	diabetes mellitus
